# Long‐Term Weight Loss Outcomes in a Virtual Weight Care Clinic Prescribing a Broad Range of Medications Alongside Behavior Change

**DOI:** 10.1002/osp4.70036

**Published:** 2025-01-08

**Authors:** Jennifer M. Clark, Brooke J. Smith, Jessie L. Juusola, Rekha B. Kumar

**Affiliations:** ^1^ Found Health, Inc. Austin Texas USA; ^2^ Anchor Outcomes LLC San Francisco California USA; ^3^ Weill Cornell Medical College New York New York USA

**Keywords:** antiobesity medication, digital health, real‐world evidence, retrospective study, weight maintenance

## Abstract

**Background:**

Virtually‐delivered obesity care has the potential to increase access to weight loss interventions at scale. While there is ample literature assessing various weight loss interventions, studies specifically demonstrating outcomes of commercial programs offering antiobesity medications in virtual care settings are lacking.

**Methods:**

This retrospective cohort study assessed the weight loss outcomes of 66,094 participants in a virtual weight care program that prescribes antiobesity medications alongside a digital behavior change program. Outcomes included the primary endpoint of percent weight loss at 12 months, as well as absolute change in body weight, change in body mass index (BMI), categorical weight loss at three, six, and 12 months, and stratifications by program engagement and medication type (first vs. second generation antiobesity medications).

**Results:**

At program enrollment, members were on average 42.6 years old and 91.5% female, with a BMI of 36.0 kg/m^2^. At 12 months, the mean percent weight loss was 8.0%, with weight loss increasing over time from 2.9 kg (SD = 3.7, Cohen's *d* = 0.8) at 3 months, to 5.8 kg (SD = 6.1, Cohen's *d* = 0.9) at 6 months, to 8.0 kg (SD = 8.7, Cohen's *d* = 0.9) at 12 months (*p* < 0.001 for all time points). At 12 months, 64.2% had achieved ≥ 5% weight loss. Weight loss outcomes increased with program engagement. At 12 months, those engaging at least once weekly lost 10.0% of body weight, while those logging weight at least weekly lost 12.0%.

**Conclusion:**

This study provides real‐world evidence that users of a virtual commercial weight care clinic who were prescribed antiobesity medications achieved clinically significant weight loss at six and 12 months. These findings support the value of virtual platforms in efficiently scaling access to high‐quality weight care.

## Graphical abstract

This retrospective study of a virtually‐delivered obesity care program found that program users lost 8% of their body weight on average at 12 months, and weight loss increased with program engagement. These findings support the value of virtual platforms in efficiently scaling access to high‐quality weight care.

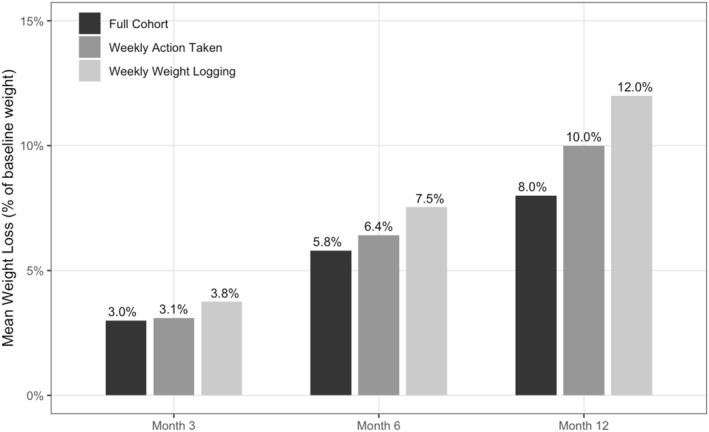

## 1 Introduction

Despite epidemiological data showing that more than 70% of adults in the United States (US) are overweight or obese, evidence suggests that fewer than 4% receive obesity care from a physician [1, 2]. Although in‐person care is the gold standard, it is not a practical option at scale as the sheer number of people with obesity across the country cannot be served by the small number of trained specialists in specific geographies [3]. Even though the number of certified obesity physicians increased by 400% from 2011 to 2019, they represent less than 1% of physicians who are certified in most regions of the US [4, 5]. These geographic limitations of care availability create major disparities in access to obesity treatment.

In addition to access considerations, high quality obesity care requires a high number of patient touch points across several disciplines (e.g., nutrition, exercise, sleep, stress management). Virtual care platforms are well‐positioned to help bridge the care accessibility gap while also allowing additional disciplines to be available to the patient through technology, both through digital tools and virtual connections to clinicians [6]. Virtual platforms allow clinicians to service patients more efficiently, reduce patient burden, and provide an alternative for people who prefer remote care. They can save patients' time and money by reducing the need for travel, wait times in clinics, and the expenses associated with an in‐person consult as well as providing options for those with limited mobility [3, 7]. Technology can facilitate providers to care for greater numbers of patients by allowing for asynchronous care and coordination, including tracking progress, providing feedback, and managing medications. Providing patients with centralized access to a wide variety of tools and specialty care to support their personalized needs shows promise to mitigate barriers to care and increase adherence with treatment modalities and improve weight loss and maintenance outcomes [3, 7, 8]. For the treatment of obesity, evidence is mounting that virtual care can be an effective modality. Not only do studies show that patients can achieve clinically significant weight loss, but virtual care can lead to weight loss outcomes comparable to in‐person care [9, 10, 11, 12, 13].

Pharmacotherapy is an important tool for obesity care, and US Food and Drug Administration (FDA) approved pharmacotherapies for obesity have been available in the US since the 1960s. However, they remain critically underutilized with only 0.8% of eligible patients receiving them from 2015 to 2018 [14]. When used as an adjuvant to lifestyle interventions, pharmacotherapy improves the probability that patients reach clinically meaningful weight loss [15]. The newest generations of antiobesity medications (i.e., semaglutide, liraglutide, tirzepatide) have recently received much attention as they are highly effective, with many patients reaching greater than 15% weight loss in clinical trials [16, 17, 18]. However, cost is a significant barrier for their use in treatment as many insurers are concerned about the magnitude of spend that wide scale coverage could entail and thus limit coverage [19]. Accordingly, it is important to ensure prescribing is done in an appropriate and focused manner so that patients are matched with the medications and weight loss tools that weigh benefits against costs [20].

Virtual care platforms provide a unique opportunity to ensure that prescribing is only done by highly trained clinicians who use an evidence‐based approach to appropriate prescribing rather than blanket prescribing of new generation medications. The ability to scale their expertise in areas such as matching patients to the right mechanism of action, administering proper dose escalation, and utilizing step therapy alongside clinical quality review, at scale across geographic regions, holds significant promise to optimize efficacy, safety, and cost outcomes. This is especially important considering that many patients need to be on medication long‐term due to evidence of weight regain when medication is discontinued [21, 22, 23, 24]. As the demand and promise for antiobesity medications have increased, many digitally‐enabled care options have become available, with several offering a mix of pharmacotherapy access alongside multi‐component behavior change programs. However, each program and its approach to care and prescribing is different, and more research is needed to evaluate the ability of these clinics to produce clinically significant outcomes across geographic regions both with regard to weight loss in the short‐term and maintenance in the long‐term.

In this study, a retrospective cohort analysis was used to assess 12‐month weight loss outcomes of a virtual commercial weight care clinic (Found Health, Inc.) that prescribes antiobesity medications alongside a comprehensive behavior change program to users across the US. Our primary objective was to examine the percent change in total body weight from the start of the program to 12 months. Additionally, we assessed absolute change in body weight, change in body mass index (BMI), and categorical weight loss (the percent of program participants achieving ≥ 5%, ≥ 10%, ≥ 15% and ≥ 20% of total body weight) at 12 months, as well as all outcomes at three and 6 months from baseline. We also analyzed clinical outcomes stratified by level of engagement with the program as well as by type of medication prescribed.

## 2 Methods

### 2.1 Study Design

A retrospective observational cohort study was used to assess weight loss outcomes of adults with overweight and obesity who self‐enrolled in Found Health, Inc. between October 2021 and October 2023. Participants in the program had to meet the following clinical requirements to be eligible to enroll: 18–65 years old; BMI ≥ 30 kg/m^2^ or ≥ 27 kg/m^2^ with at least one comorbidity (e.g., diabetes, hypertension, dyslipidemia); not pregnant, breastfeeding, or trying to conceive; at least one previous weight loss attempt; and no medical contraindications. To be included in the analysis, participants had to have reported their height and weight at baseline, been prescribed at least one antiobesity medication during the study period, and had weight records at one or more follow‐up time points. All participants were US residents. Individuals were excluded from the cohort if they reported height, weight, or BMI as outliers at baseline (start of program) using the following criteria for biologically implausible values: (1) height: < 122 cm (48 inches) or > 213 cm (84 inches), (2) weight: < 34 kg (75 pounds) or > 318 kg (700 pounds), or (3) BMI: ≥ 100 kg/m^2^ [25, 26]. Individuals were also excluded from analysis if they recorded biologically implausible weight records or ones that indicated BMI change was ≥ 10.5 kg/m^2^, ≥ 21.0 kg/m^2^, and ≥ 42.0 kg/m^2^ at three, six, and 12 months from baseline, respectively [27].

All data used were de‐identified data previously collected for commercial purposes, and thus the study was deemed exempt from ethics approval by the WCG Institutional Review Board (#1‐1688560‐1).

### 2.2 Program Description

Found Health, Inc. is a virtual commercial weight care program that provides mobile‐ and web‐based services through a paid subscription, which includes an app‐based multicomponent behavior change program, asynchronous access to a health coach, behavior change content and tracking tools, and access to an online support community. The behavior change program is participant‐led, which means that program users are encouraged to engage in the ways that feel most supportive for reaching their weight loss goals. Options for engagement include personalized guidance from the app and coach, based on cognitive behavioral therapy techniques, nutrition guidance, and content and recommendations regarding incorporating physical activity and managing the psychological and social elements that can contribute to weight loss (e.g., stress management, optimizing sleep, and engaging with peers). Guidance for nutrition is not oriented around caloric restriction alone, but rather balancing food choices and understanding how to make conscious decisions around meal planning. The app supports users by allowing them to log weight and various health behaviors, making the logs visible to their coach to encourage more active dialog, and providing an interface to engage with a supportive community. App content and coaching expertise span several disciplines, including nutrition, exercise physiology, and diabetes education, and accordingly, program design is supported by expertise across these disciplines. Program members continue to have access to the app for logging and community involvement after ending their paid subscription, as these tools can support a holistic approach to weight management. Eligible program members also receive care through digital consultations with Found‐affiliated clinicians trained in obesity medicine.

Membership costs for members eligible for medication include the cost of certain prescribed antiobesity medications, with the exception of glucagon‐like peptide 1 agonists (GLP‐1s) and GLP‐1/glucose‐dependent insulinotropic polypeptide dual agonists (GLP‐1/GIPs), referred to collectively as GLP‐1 medications. Members prescribed GLP‐1s cover the cost of the medication through their own health insurance plan or out‐of‐pocket. Clinicians make prescription recommendations based on the patient's phenotype (a characterization based on hunger and fullness patterns, eating behaviors, and degree of obesity), potential risks, medical history and comorbidities, previous experiences, behavioral factors, member preferences, supply chain availability, and cost to the participant. At any point during their subscription, eligible members are able to request to switch medications or discontinue their use through a clinician consultation.

### 2.3 Measures

#### 2.3.1 Body Weight and BMI

Program users can record their body weight in multiple ways. Baseline weight was self‐reported in a questionnaire administered prior to program enrollment. After enrolling in the program, users can self‐report weight by logging it in the program app or web portal, or they can sync the program app with their native mobile health tracking app on their phone (e.g., Apple Health, Samsung Health, Google Fit). This allows any weight they may log in those apps or that is synced to those apps from a connected digital scale to flow into the program app, although during the study period they were required to actively allow each weight log from a synced app. They also self‐report weight in a questionnaire at any time they request a medication refill.

Baseline weight was calculated as the mean of all weight measurements on the day with at least one weight measurement closest to the program start date within a window of ± 7 days. Follow‐up weights at three, six, and 12 months were calculated as the mean of all weight measurements on the day with at least one weight measurement closest to the month of follow‐up within the window of ± 30 days. Absolute change in weight and percentage change in weight were calculated at the individual level and then the average of each metric was calculated to determine population level means. To calculate BMI, height reported on program enrollment questionnaires was used, and obesity level was categorized based on BMI levels.

Maintenance of weight loss was calculated for those who reached at least 3% weight loss at month three or six and had month 12 weight data as well. Those whose weight at month 12 was no more than 3% higher than their lowest value, whether that was at month three or month six, were considered to have maintained their weight loss. Thus, the maintenance metric includes those who continued to lose weight after month six. A threshold of 3% to define maintenance was selected based on previously published recommendations and methodologies [28, 29].

### 2.4 Program User Characteristics and Medications

Self‐reported demographic information including age at program start date, biological sex at birth, and state of residence was extracted from program enrollment questionnaires. The state of residence was used to determine the participant's US census region to characterize geographical trends [30]. Program members also self‐reported their medical history and comorbidities at program enrollment.

Antiobesity medications were prescribed during clinical consultations, and information about these prescriptions was recorded in an electronic health record. Data recorded included medication name, days' supply, dosage, and formulation. Program members typically receive prescriptions for 35‐ or 90‐days’ supply, and can request a refill or discuss switching medications with the clinical staff as needed.

Clinical outcomes were stratified by whether GLP‐1 medication was prescribed through the program. The population with 12‐month data was classified into two groups: no prescriptions for GLP‐1 medication during the 12‐month period or six or more prescriptions for GLP‐1 medications. Prescriptions for GLP‐1 medications typically include 28 days of supply, so six prescriptions signifies approximately 6 months of supply. Those who had at least one but less than six prescriptions for GLP‐1 medications were excluded from this subgroup analysis.

### 2.5 Program Engagement

Program engagement was measured in two ways: (1) by the number of days per week with proactive actions taken by the user (e.g., logging a behavior in the app, engaging with a coach or clinician, reading an educational article, engaging with the community), and (2) by the number of weight logs per week and per month. Mean active days per week and mean weight logs per week and month were calculated for each follow‐up time period cohort (e.g., 3‐month cohort metrics were calculated over each user's 3‐month time period, plus 30 days to match the buffer for weight data inclusion). Two engaged subgroups were defined in order to stratify clinical outcomes by engagement. These were based on (1) at least one active day per week on average and (2) logging weight at least once per week on average.

### 2.6 Statistical Analysis

To test for differences in the distribution of baseline characteristics between mutually exclusive groups, *t*‐tests and chi‐square tests were used for continuous and categorical variables, respectively. For the full cohort and for all subgroups (stratifications by engagement and GLP‐1 medication prescriptions), differences in weight outcomes, both in terms of kg and BMI, from baseline to each follow‐up time point were evaluated using two‐sided paired t‐tests for significance and Cohen's *d* for effect size interpretation. Descriptive statistics were used to measure categorical weight loss and change in BMI category. Two‐sided two‐sample t‐tests were used to evaluate the differences in weight outcomes between the ≥ 6 GLP‐1 group and those having no GLP‐1 prescriptions by month 12. To assess whether the baseline factors of age, sex, US region, and BMI (≥ 35) related to percent change in weight at 12 months, we fit multivariable linear models adjusting for these characteristics. The most parsimonious model containing significant predictors was selected as the final model. As a sensitivity analysis to ensure the robustness of findings, we ran linear mixed‐effects models (LMMs) using the study population with at least one follow‐up weight record to estimate the average weight change at each follow‐up time point. We ran an unadjusted model containing fixed effects for time (as a categorical variable at study time points), random intercepts for repeated measures across subjects, and random slopes for study time points. In addition, we ran an adjusted model that included fixed effects for age at baseline and sex. Both LMMs used an unstructured covariate matrix, and model fit was evaluated using Akaike Information Criterion (AIC) and Bayesian Information Criterion (BIC) and residual plots. For ease of interpretation and comparison, the unadjusted model was selected as the final model due to weight change estimates being nearly identical in the unadjusted and adjusted models. All analyses were conducted in R (version 4.3.2).

## 3 Results

### 3.1 Member Characteristics

**FIGURE 1:**
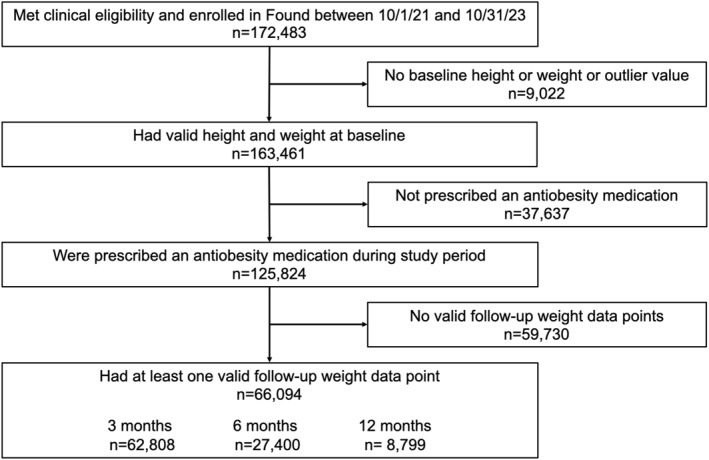
Member enrollment and study participation flow chart.

A total of 125,824 individuals enrolled in the Found program during the study period, had qualifying baseline weight and height, and were prescribed an antiobesity medication (Figure 1). Of those, 66,094 had a valid weight record for at least one follow‐up time point, with 8799 having 12‐month weight data (Figure 1). The study population was 91.5% female, had a mean age of 42.6 years, and were distributed geographically across the US, with 40.6% of the cohort residing in the South (Table 1). Mean weight at baseline was 99.6 kg (SD = 20.6) and mean BMI was 36.0 kg/m^2^ (SD = 6.5) (Table 1). Nearly half (47.9%) had a BMI of 35 or higher, and 12.2% reported having pre‐diabetes (Table 1).

**TABLE 1:** Baseline demographic and clinical characteristics for the full cohort.

Member characteristic at baseline	Full cohort *n* = 66,094
Age (y), mean (SD)	42.6 (9.6)
Biological sex at birth, *n* (%)	
Female	60,449 (91.5)
Male	5628 (8.5)
Intersex	2 (< 0.1)
Unknown	15 (< 0.1)
Geographical region, *n* (%)	
Northeast	9040 (13.7)
Midwest	14,296 (21.6)
South	26,845 (40.6)
West	15,908 (24.1)
Unknown	5 (< 0.1)
Weight (kg), mean (SD)	99.6 (20.6)
BMI (kg/m^2^), mean (SD)	36.0 (6.5)
BMI category, *n* (%)	
Overweight: 25.0–29.9 kg/m^2^	9025 (13.7)
Class 1 obesity: 30.0–34.9 kg/m^2^	25,408 (38.4)
Class 2 obesity: 35.0–39.9 kg/m^2^	16,786 (25.4)
Class 3 obesity: ≥ 40.0 kg/m^2^	14,875 (22.5)
Comorbidities, *n* (%)	
Anxiety	14,476 (21.9)
Arthritis	12,117 (18.3)
Body pain	36,852 (55.8)
Depression	17,688 (26.8)
Dyslipidemia	13,765 (20.8)
Hypertension	13,818 (20.9)
Metabolic dysfunction‐associated steatotic liver disease (MASLD)	4918 (7.4)
Obstructive sleep apnea	9334 (14.1)
Polycystic ovary syndrome (PCOS)	6793 (10.3)
Pre‐diabetes	8064 (12.2)
Type II diabetes	1482 (2.2)

Abbreviations: BMI, body mass index; SD, standard deviation.

### 3.2 Program Engagement

On average across the time periods with follow‐up weight data, program users proactively engaged with the program 1.7 days per week and logged their weight 2.6 times per month. For those with 12‐month data, average days of engagement with the program per week was 1.6 and average monthly weight records was 2.9. Of this cohort, 54.3% engaged with the program at least once per week on average, and 20.2% logged their weight at least once per week on average. Baseline demographic and clinical characteristics of these engaged subgroups can be found in Supplemental Table S1.

### 3.3 Clinical Outcomes

**FIGURE 2:**
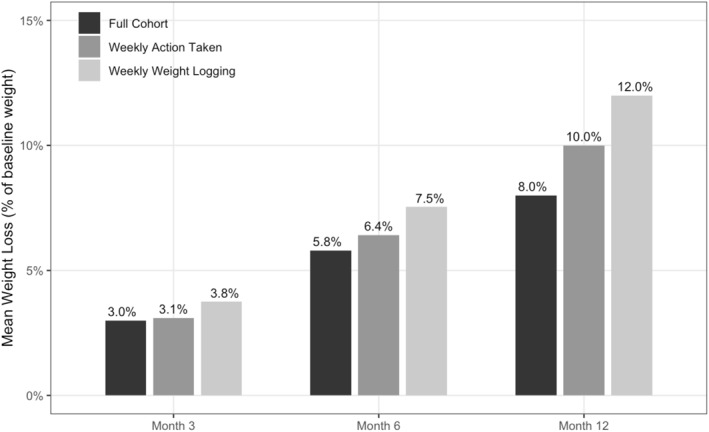
Mean percent reduction in total body weight at three, six, and 12 months for the full cohort and engaged cohorts.

Program users demonstrated significant reductions in weight from baseline at all time points analyzed, with mean percent weight loss from baseline of 8.0% (SD = 8.3%) at 12 months (Figure 2, Table 2). Weight loss increased over time, with mean weight loss from baseline increasing from 2.9 kg (SD = 3.7, Cohen's *d* = 0.8) at 3 months to 5.8 kg (SD = 6.1, Cohen's *d* = 0.9) at 6 months to 8.0 kg (SD = 8.7, Cohen's *d* = 0.9) at 12 months (Table 2, *p* < 0.001 for all time points). Decreases in BMI followed a similar trend, with a mean decrease from baseline of 1.1 kg/m^2^ (SD = 1.3, Cohen's *d* = 0.8) at 3 months to 2.1 kg/m^2^ (SD = 2.2, Cohen's *d* = 1.0) at 6 months and to 2.9 kg/m^2^ (SD = 3.1, Cohen's *d* = 0.9) at 12 months (Table 2, *p* < 0.001 for all time points). In the final multivariable model adjusting for baseline factors, results were consistent with unadjusted estimates, with a baseline BMI of 35 or higher having the only notable effect size (correlating with a 1.3% increase in weight loss at 12 months). For the sensitivity analysis using linear mixed‐effects modeling to measure our primary outcomes of interest, weight loss results were similar to those observed in the primary analysis using paired t‐tests, with estimates slightly attenuated but still highly significant at months 6 and 12 (Supplemental Table S2).

**TABLE 2:** Body weight and BMI outcomes at three, six, and 12 months from baseline for the full cohort.

Mean (SD)	3 months (*n* = 62,808)	6 months (*n* = 27,400)	12 months (*n* = 8799)
Weight at baseline (kg)	99.6 (20.6)	99.3 (20.5)	98.5 (20.3)
Weight at follow‐up (kg)	96.6 (20.3)	93.5 (20.2)	90.6 (20.0)
Decrease in weight (kg)	2.9 (3.7)	5.8 (6.1)	8.0 (8.7)
*p*‐value from baseline	< 0.001	< 0.001	< 0.001
BMI at baseline (kg/m^2^)	36.0 (6.5)	35.9 (6.5)	35.7 (6.3)
BMI at follow‐up (kg/m^2^)	35.0 (6.4)	33.8 (6.4)	32.8 (6.4)
Decrease in BMI (kg/m^2^)	1.1 (1.3)	2.1 (2.2)	2.9 (3.1)
*p*‐value from baseline	< 0.001	< 0.001	< 0.001

Abbreviations: BMI, body mass index; SD, standard deviation.

**FIGURE 3:**
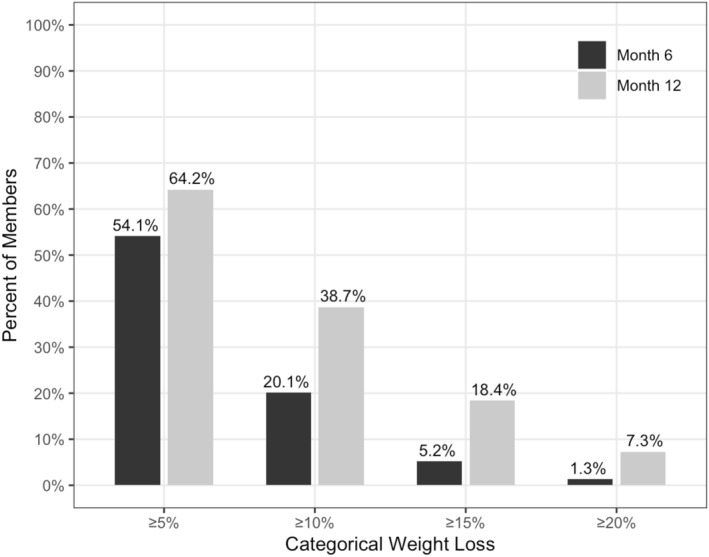
Categorical weight loss outcomes at six and 12 months.

Clinically significant weight loss, defined as 5% or greater weight loss, was achieved by 54.1% of users at 6 months, and by 64.2% at 12 months (Figure 3). At 12 months, 18.4% of users had lost 15% or more of their weight, and 7.3% had achieved 20% or greater weight loss (Figure 3). Nearly half of the users (48.6%) reduced their BMI enough to move down at least one BMI category by 12 months, while 6.5% moved down two BMI categories.

For those program users with month 12 weight data who had at least 3% weight loss by month three or month six (*n* = 6191), 82.9% (*n* = 5131) were considered to have maintained weight loss, with weight at month 12 being no more than 3% higher than their lowest value at month three or six. Of those who maintained weight loss, 79.6% (*n* = 4083) lost additional weight at 12 months.

Weight loss was greater in the engaged subgroups. Average weight loss at 12 months was 10.0% for the 54.3% of users who engaged with the program at least once per week on average, and 12.0% for the 20.2% who logged their weight at least once per week on average (Figure 2). The decrease in BMI at 12 months was 3.6 kg/m^2^ (SD = 3.0) for those who engaged with the program weekly on average, and 4.4 kg/m^2^ (SD = 2.9) for those who logged weight at least weekly on average (Table 3, *p* < 0.001 for both subgroups). Of those who engaged weekly on average, 75.3% achieved clinically significant weight loss of 5% or greater at 12 months, and 84.7% of those who logged weight weekly on average did the same.

**TABLE 3:** Body weight and BMI outcomes at 12 months for the engaged cohorts and subgroups stratified by GLP‐1 prescriptions.

Mean (SD)	Engaged cohort: Weekly action taken (*n* = 4778)	Engaged cohort: Weekly weight logged (*n* = 1773)	≥ 6 GLP‐1 prescriptions (*n* = 265)	No GLP‐1 prescriptions (*n* = 7966)
Weight at baseline (kg)	99.3 (20.5)	100.0 (20.1)	98.1 (17.9)	98.5 (20.3)
Weight at follow‐up (kg)	89.3 (19.7)	87.9 (18.8)	84.5 (17.5)	90.5 (20.0)
Decrease in weight (kg)	10.0 (8.1)	12.1 (8.0)	13.6 (8.3)^a^	7.9 (8.6)
*p*‐value from baseline	< 0.001	< 0.001	< 0.001	< 0.001
BMI at baseline (kg/m^2^)	36.1 (6.6)	36.3 (6.6)	35.4 (5.9)	35.7 (6.4)
BMI at follow‐up (kg/m^2^)	32.5 (6.4)	31.9 (6.2)	30.5 (5.8)	32.8 (6.4)
Decrease in BMI (kg/m^2^)	3.6 (3.0)	4.4 (2.9)	4.9 (3.0)^a^	2.9 (3.1)
*p*‐value from baseline	< 0.001	< 0.001	< 0.001	< 0.001

Abbreviations: BMI, body mass index; GLP‐1, glucagon‐like peptide 1 agonist medication; SD, standard deviation.

^a^ *p*‐value between GLP‐1 prescription groups for change in weight and change in BMI is < 0.001.

**FIGURE 4:**
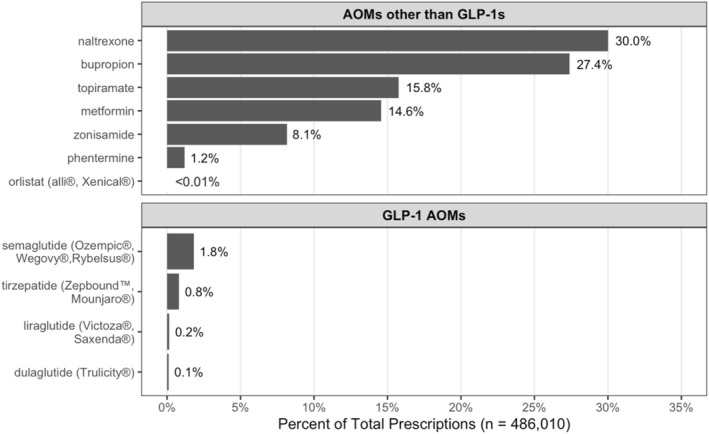
Antiobesity medication (AOM) prescription breakdown for the analytic sample during the study period. Some medications were only available for a portion of the study period. Many members were prescribed medications in combination.

During the study period, 88% of antiobesity medication prescriptions written for members in the study population were for four common generic medications (Figure 4). Only 2.9% of prescriptions were for GLP‐1 medications. When those with 12‐month data were stratified by whether they had prescriptions for GLP‐1 medications, 3% (*n* = 265) had six or more prescriptions during the 12‐month period. This subgroup had significantly greater weight loss than those who had no prescriptions for GLP‐1 medication, with 13.9% weight loss as compared to 7.9% (13.6 vs. 7.9 kg, *p* < 0.001, Table 3). The majority of the subgroup, 90.6%, achieved clinically significant weight loss of 5% at 12 months, and 20.0% lost 20% or more of their body weight. Baseline demographic and clinical characteristics of the stratified subgroups can be found in Supplemental Table S3.

## 4 Discussion

In this real‐world study, Found users with weight log data achieved clinically meaningful and statistically significant average weight loss at six and 12 months, with an average weight loss of 8% at 12 months. Nearly two‐thirds of the population achieved clinically meaningful weight loss at 12 months, and over 80% of those who lost 3% or more of baseline body weight by three or 6 months maintained the weight loss or lost additional weight. This level of sustained weight loss in those with weight log data is noteworthy as weight regain at 1 year is common after weight loss programs [31, 32]. The large population studied here, of over 66,000 total, and nearly 9000 with 12‐month data, not only provides confidence in the statistical findings of this analysis but also highlights the ability of virtual weight care programs to drive weight loss and maintenance at scale. The member population was mostly female, which is typical of those who engage with weight loss programs, and had broad geographic representation across the US, again showcasing the value of virtual care in broadening access to high quality weight care. Baseline BMI at program enrollment was 36.0 kg/m^2^ and nearly half had a BMI of 35 kg/m^2^ or higher, signifying that the program has been successful at engaging those most likely to have the greatest clinical benefits from losing weight.

Weight loss results were greater in those who had higher engagement with the program, which is consistent with results seen with other digital weight loss interventions [33, 34, 35]. Average weight loss at 12 months was 8% for the overall population, 10% in those who engaged with the program at least weekly on average, and 12% for those who logged their weight weekly on average. Digital programs can provide comprehensive programming, tools, and access to multiple forms of real‐time support to increase engagement that is challenging to achieve with standalone prescribing or diet and exercise guidance. Since engagement is driven by the components and design of the platform itself, programs must be evaluated independently for their effectiveness. To our knowledge, this study is the first to provide real‐world evidence that a virtual multicomponent commercial program prescribing a broad range of antiobesity medications can lead to clinically significant weight loss for a large, generalizable population seeking medically‐directed digital weight loss solutions and willing to log weight over time.

The vast majority of antiobesity medications prescribed in this population are medications that have been available for decades and are low‐cost, as opposed to the newly available and high‐cost GLP‐1 medications. While average weight loss outcomes were meaningful regardless of the type of medication prescribed, those who received 6 months' worth of GLP‐1 medication prescriptions lost more weight than those not receiving GLP‐1s; this result is expected, and GLP‐1 outcomes are in line with other published studies [16, 36]. However, while GLP‐1 medications have been shown to be very effective at driving weight loss, they are not effective for all individuals, cost orders of magnitude more than other antiobesity medications, and have been shown to require long‐term use, with weight rebounding when stopped [23, 24]. The need for long‐term use is especially problematic given the cost of these medications. Budgetary concerns about widespread GLP‐1 use for obesity management are not unfounded, and as such, one must consider the value of achieving 8% weight loss in a very large population at a low cost as compared to 14% weight loss in a much smaller population at a very high cost.

The ability to achieve meaningful weight loss and maintenance in a large population with predominantly low‐cost medications enforces the value of facilitating prescribing of a broad range of antiobesity medications tailored to a patient's phenotype (biological and behavior factors), medical history, tolerability, and preferences. Regardless of the medication type prescribed in this study, the weight loss target recommended by the American College of Cardiology, American Heart Association, and The Obesity Society of ≥ 5% total body weight was achieved by the majority of the study population, and a substantial proportion reached ≥ 10% [37]. Importantly, this level of weight loss can lead to improvement in several obesity‐related comorbidities, such as type 2 diabetes, hypertension, dyslipidemia, cardiovascular disease, metabolic dysfunction‐associated steatotic liver disease, and health‐related quality of life [38]. Even > 2.5% weight loss can improve a variety of psychological parameters, eating disorders, and quality of life [39].

This study had a number of limitations, as is typical of studies conducted with real‐world data. First, during the study period, subscriptions were self‐paid, and as such, this sample may be more motivated than a population who had the program provided as a benefit from an employer or insurance company. Second, the one‐arm retrospective design did not allow for direct comparison with a control group. In the absence of a direct control group, we can look to benchmark weight loss expected in various settings. The results seen in this study are in range with studies that assess weight loss outcomes from antiobesity medication prescribed at academic centers, community programs, and specialist clinics [15, 40]. Importantly, weight loss in this study is higher than what is typically seen in the absence of a weight loss program [41, 42]. Third, some of the weight data used for clinical outcome analysis was self‐reported, which leaves the possibility of user error in reporting. To combat this, biologically implausible values and inconsistent weight records were excluded from the dataset using previously published methods. Additionally, because weight logging is often a key part of how an individual tracks their own progress, we expect that users often quality checked their own data. This is consistent with studies that have shown high accuracy of self‐reported weight for participants in weight loss programs [43]. Lastly, we were limited by the data that was available through the program itself. Race, ethnicity, and other socioeconomic factors such as educational level and annual income were not collected by the program, limiting the ability to characterize the study population. Additionally, we were only able to assess outcomes for those who recorded a weight value at a follow‐up time point. In this study, 53% of those who were prescribed an antiobesity medication during the study period had at least one valid follow‐up weight data point. This is higher than is typically seen in retrospective cohort analyses of digital health programs, which often have follow‐up data for fewer than 20% of eligible program members [27, 44]. Nevertheless, it is important to understand the potential for bias when analysis is limited to a subset of program users. In order to explore whether those with follow‐up weight data differed from those without, we compared baseline demographic and clinical characteristics between the groups. Due to the very large sample sizes, we observed statistically significant differences between the groups on factors such as age, sex, and region, despite small, often negligible, absolute differences (Supplemental Table S4).

Despite the limitations that come with analyzing real‐world data, it is important to assess the real‐world impact of this program, and this study has notable strengths. A key feature is the large sample size with representation across the geographic regions of the US, which demonstrates the ability of the program to drive care accessibility. The 12‐month time frame and interim time points analyzed allow us to present both a long‐term view of program outcomes as well as the progression to the 12‐month results. This analysis also includes a broad range of antiobesity medication usage, which provides a real‐world assessment of what can be achieved with broader access to prescribing by clinicians trained in obesity medicine, rather than blanket prescribing of medications based on patient demand. Another strength in this study is the quality of the clinical program being assessed, as one that has implemented high quality safety protocols, uses dose escalation for medications, and places a high standard on clinical quality review processes. Overall, this study shows that virtual solutions with clinicians trained in obesity medicine can be successfully delivered at scale across geographic regions.

In conclusion, this study provides real‐world evidence that medically‐directed, virtual commercial weight care that pairs antiobesity medication alongside a comprehensive behavior change program can play an important role in treating overweight and obesity at scale. Weight maintenance is a significant challenge for both patients and clinicians, and long‐term behavioral support and use of antiobesity medications are critical to managing weight plateaus and weight regain. As obesity rates continue to rise, it is important to continue to deliver and improve upon solutions that are medically‐guided with responsible prescribing and long‐term care and support, and to build a real‐world evidence base around the impact of these solutions. Future studies should include control groups in order to facilitate examination of a causal relationship between program use and outcomes. Additional areas of future exploration include how to utilize GLP‐1s most effectively versus other medications that are lower in cost and may be less vulnerable to supply shortages, as well as to better understand the clinical and long‐term value of slow, sustained weight loss as compared to rapid weight loss with weight regain.

## Conflicts of Interest

J.M.C. declares she is a consultant for Found Health Inc. and a former employee who received salary, stock options and support for conference travel. B.J.S. declares she is employed by Found Health Inc. and receives salary and stock options. J.L.J. declares she receives consulting fees from Found Health Inc. R.B.K. declares she is employed by Found Health Inc. and receives salary, stock options, and support for travel to meetings. R.B.K. declares she is also on the Board of Directors for Duke Global Health Institute, is a consultant for Novo Nordisk and Eli Lilly, is a shareholder in Vivus, and receives honoraria to speak at the Blackburn Obesity Course.

## Supporting information

Supporting Information S1

## References

[1] National Center for Health Statistics , Selected Health Conditions and Risk Factors, by Age: United States, Selected Years 1988–1994 Through 2017–2018, (Atlanta, GA, USA: US Centers for Disease Control and Prevention, 2019).

[2] A. Stokes , J. M. Collins , B. F. Grant , et al., “Prevalence and Determinants of Engagement With Obesity Care in the United States,” Obesity 26, no. 5 (2018): 814–818, 10.1002/oby.22173. 29626388 PMC5947584

[3] S. Kahan , M. Look , and A. Fitch , “The Benefit of Telemedicine in Obesity Care,” Obesity 30, no. 3 (2022): 577–586, 10.1002/oby.23382. 35195367

[4] K. A. Gudzune , V. R. Johnson , C. T. Bramante , and F. C. Stanford , “Geographic Availability of Physicians Certified by the American Board of Obesity Medicine Relative to Obesity Prevalence,” Obesity 27, no. 12 (2019): 1958–1966, 10.1002/oby.22628. 31515965 PMC6868336

[5] C. C. Pollack , T. Onega , J. A. Emond , et al., “A National Evaluation of Geographic Accessibility and Provider Availability of Obesity Medicine Diplomates in the United States Between 2011 and 2019,” International Journal of Obesity 46, no. 3 (2022): 669–675, 10.1038/s41366-021-01024-9. 34992242 PMC8881297

[6] T. Wadden , J. Tronieri , and M. Butryn , “Lifestyle Modification Approaches for the Treatment of Obesity in Adults,” American Psychologist 75, no. 2 (2020): 235–251, 10.1037/amp0000517. 32052997 PMC7027681

[7] H. K. Y. Almathami , K. T. Win , and E. Vlahu‐Gjorgievska , “Barriers and Facilitators That Influence Telemedicine‐Based, Real‐Time, Online Consultation at Patients’ Homes: Systematic Literature Review,” Journal of Medical Internet Research 22, no. 2 (2020): e16407, 10.2196/16407. 32130131 PMC7059083

[8] N. Hinchliffe , M. S. Capehorn , M. Bewick , and J. Feenie , “The Potential Role of Digital Health in Obesity Care,” Advances in Therapy 39, no. 10 (2022): 4397–4412, 10.1007/s12325-022-02265-4. 35925469 PMC9362065

[9] K. Ufholz and D. Bhargava , “A Review of Telemedicine Interventions for Weight Loss,” Curr Cardiovasc Risk Rep 15, no. 9 (2021): 17, 10.1007/s12170-021-00680-w. 34306296 PMC8280385

[10] M. Al‐Badri , C. L. Kilroy , J. I. Shahar , et al., “In‐person and Virtual Multidisciplinary Intensive Lifestyle Interventions Are Equally Effective in Patients With Type 2 Diabetes and Obesity,” Ther Adv Endocrinol Metab 13 (2022): 20420188221093220, 10.1177/20420188221093220. 35464878 PMC9019312

[11] M. L. Griebeler , W. S. Butsch , P. Rodriguez , et al., “The Use of Virtual Visits for Obesity Pharmacotherapy in Patients With Overweight or Obesity Compared With In‐Person Encounters,” Obesity 30, no. 11 (2022): 2194–2203, 10.1002/oby.23548. 36156456 PMC9826334

[12] B. G. Tchang , C. Morrison , J. T. Kim , et al., “Weight Loss Outcomes With Telemedicine During COVID‐19,” Frontiers in Endocrinology 13 (2022): 793290, 10.3389/fendo.2022.793290. 35360066 PMC8960113

[13] S. Rajkumar , E. Davidson , M. Bell , et al., “Effect of Telehealth‐Based Versus In‐person Nutritional and Exercise Intervention on Type II Diabetes Mellitus Improvement and Efficiency of Human Resources Utilization in Patients With Obesity,” Obes Sci Pract 9, no. 5 (2023): 468–476, 10.1002/osp4.667. 37810527 PMC10551111

[14] J. MacEwan , H. Kan , K. Chiu , J. L. Poon , S. Shinde , and N. N. Ahmad , “Antiobesity Medication Use Among Overweight and Obese Adults in the United States: 2015‐2018,” Endocrine Practice 27, no. 11 (2021): 1139–1148, 10.1016/j.eprac.2021.07.004. 34265455

[15] G. Calderon , D. Gonzalez‐Izundegui , K. L. Shan , et al., “Effectiveness of Anti‐Obesity Medications Approved for Long‐Term Use in a Multidisciplinary Weight Management Program: A Multi‐Center Clinical Experience,” International Journal of Obesity 46, no. 3 (2022): 555–563, 10.1038/s41366-021-01019-6. 34811486 PMC8881310

[16] X. Pi‐Sunyer , A. Astrup , K. Fujioka , et al., “A Randomized, Controlled Trial of 3.0 Mg of Liraglutide in Weight Management,” New England Journal of Medicine 373, no. 1 (2015): 11–22, 10.1056/NEJMoa1411892. 26132939

[17] J. P. H. Wilding , R. L. Batterham , S. Calanna , et al., “Once‐Weekly Semaglutide in Adults With Overweight or Obesity,” New England Journal of Medicine 384, no. 11 (2021): 989–1002, 10.1056/NEJMoa2032183. 33567185

[18] A. M. Jastreboff , L. J. Aronne , N. N. Ahmad , et al., “Tirzepatide Once Weekly for the Treatment of Obesity,” New England Journal of Medicine 387, no. 3 (2022): 205–216, 10.1056/NEJMoa2206038. 35658024

[19] Insurer strategies to control costs associated with weight loss drugs , Peterson‐KFF Health System Tracker, (San Francisco, CA, USA: KFF, n.d.): https://www.healthsystemtracker.org/brief/insurer‐strategies‐to‐control‐costs‐associated‐with‐weight‐loss‐drugs/. Accessed, August 22, 2024.

[20] D. D. Kim , J. H. Hwang , and A. M. Fendrick , “Balancing Innovation and Affordability in Anti‐obesity Medications: The Role of an Alternative Weight‐Maintenance Program,” Health Aff Sch 2, no. 6 (2024), 10.1093/haschl/qxae055. PMC11138958 38828004

[21] T. A. Wadden , P. Hollander , S. Klein , et al., “Weight Maintenance and Additional Weight Loss With Liraglutide After Low‐Calorie‐Diet‐Induced Weight Loss: The SCALE Maintenance Randomized Study,” International Journal of Obesity 37, no. 11 (2013): 1443–1451, 10.1038/ijo.2013.120. 23812094

[22] D. Rubino , N. Abrahamsson , M. Davies , et al., “Effect of Continued Weekly Subcutaneous Semaglutide vs Placebo on Weight Loss Maintenance in Adults With Overweight or Obesity,” JAMA 325, no. 14 (2021): 1–12, 10.1001/jama.2021.3224. PMC7988425 33755728

[23] J. P. H. Wilding , R. L. Batterham , M. Davies , et al., “Weight Regain and Cardiometabolic Effects After Withdrawal of Semaglutide: The STEP 1 Trial Extension,” Diabetes, Obesity and Metabolism 24, no. 8 (2022): 1553–1564, 10.1111/dom.14725. PMC9542252 35441470

[24] L. J. Aronne , N. Sattar , D. B. Horn , et al., “Continued Treatment With Tirzepatide for Maintenance of Weight Reduction in Adults With Obesity: The SURMOUNT‐4 Randomized Clinical Trial,” JAMA 331, no. 1 (2024): 38–48, 10.1001/jama.2023.24945. 38078870 PMC10714284

[25] J. Y. Breland , C. S. Phibbs , K. J. Hoggatt , et al., “The Obesity Epidemic in the Veterans Health Administration: Prevalence Among Key Populations of Women and Men Veterans,” Journal of General Internal Medicine 32, no. S1 (2017): 11–17, 10.1007/s11606-016-3962-1. PMC5359156 28271422

[26] S. H. Chan and S. D. Raffa , “Examining the Dose‐Response Relationship in the Veterans Health Administration’s MOVE!® Weight Management Program: A Nationwide Observational Study,” Journal of General Internal Medicine 32, no. S1 (2017): 18–23, 10.1007/s11606-017-3992-3. 28271425 PMC5359164

[27] H. H. Kim , Y. Kim , A. Michaelides , and Y. R. Park , “Weight Loss Trajectories and Related Factors in a 16‐Week Mobile Obesity Intervention Program: Retrospective Observational Study,” Journal of Medical Internet Research 24, no. 4 (2022): e29380, 10.2196/29380. 35436211 PMC9055473

[28] M. Weintraub , D. D’Angelo , B. Tchang , et al., “Five‐Year Weight Loss Maintenance With Obesity Pharmacotherapy,” Journal of Clinical Endocrinology & Metabolism 108, no. 9 (2023): e832–e841, 10.1210/clinem/dgad100. 36810608 PMC10438886

[29] J. Stevens , K. P. Truesdale , J. E. McClain , and J. Cai , “The Definition of Weight Maintenance,” International Journal of Obesity 30, no. 3 (2006): 391–399, 10.1038/sj.ijo.0803175. 16302013

[30] U.S. Census Bureau , Census Regions and Divisions of the United States, (Washington, DC, USA: US Census Bureau, n.d.).

[31] J. W. Anderson , E. C. Konz , R. C. Frederich , and C. L. Wood , “Long‐Term Weight‐Loss Maintenance: A Meta‐Analysis of US Studies,” American Journal of Clinical Nutrition 74, no. 5 (2001): 579–584, 10.1093/ajcn/74.5.579. 11684524

[32] K. D. Hall and S. Kahan , “Maintenance of Lost Weight and Long‐Term Management of Obesity,” Medical Clinics of North America 102, no. 1 (2018): 183–197, 10.1016/j.mcna.2017.08.012. 29156185 PMC5764193

[33] S. L. Painter , R. Ahmed , J. O. Hill , et al., “What Matters in Weight Loss? an In‐Depth Analysis of Self‐Monitoring,” Journal of Medical Internet Research 19, no. 5 (2017): e160, 10.2196/jmir.7457. 28500022 PMC5446667

[34] A. Carey , Q. Yang , L. DeLuca , T. Toro‐Ramos , Y. Kim , and A. Michaelides , “The Relationship Between Weight Loss Outcomes and Engagement in a Mobile Behavioral Change Intervention: Retrospective Analysis,” JMIR Mhealth Uhealth 9, no. 11 (2021): e30622, 10.2196/30622. 34747706 PMC8663454

[35] M. L. Patel , L. N. Wakayama , and G. G. Bennett , “Self‐Monitoring via Digital Health in Weight Loss Interventions: A Systematic Review Among Adults With Overweight or Obesity,” Obesity 29, no. 3 (2021): 478–499, 10.1002/oby.23088. 33624440 PMC12838191

[36] W. Ghusn , A. De la Rosa , D. Sacoto , et al., “Weight Loss Outcomes Associated With Semaglutide Treatment for Patients With Overweight or Obesity,” JAMA Network Open 5, no. 9 (2022): e2231982, 10.1001/jamanetworkopen.2022.31982. 36121652 PMC9486455

[37] M. D. Jensen , D. H. Ryan , C. M. Apovian , et al., “2013 AHA/ACC/TOS Guideline for the Management of Overweight and Obesity in Adults: A Report of the American College of Cardiology/American Heart Association Task Force on Practice Guidelines and the Obesity Society,” Circulation 129, no. 25_suppl_2 (2014): S102–S138, 10.1161/01.cir.0000437739.71477.ee. 24222017 PMC5819889

[38] D. B. Horn , J. P. Almandoz , and M. Look , “What Is Clinically Relevant Weight Loss for Your Patients and How Can it Be Achieved? A Narrative Review,” Postgraduate Medical Journal 134, no. 4 (2022): 359–375, 10.1080/00325481.2022.2051366. 35315311

[39] Z. Pataky , I. Carrard , V. Gay , et al., “Effects of a Weight Loss Program on Metabolic Syndrome, Eating Disorders and Psychological Outcomes: Mediation by Endocannabinoids?,” Obesity Facts 11, no. 2 (2018): 144–156, 10.1159/000487890. 29631275 PMC5981584

[40] N. N. Ahmad , S. Robinson , T. Kennedy‐Martin , J. L. Poon , and H. Kan , “Clinical Outcomes Associated With Anti‐Obesity Medications in Real‐World Practice: A Systematic Literature Review,” Obesity Reviews 22, no. 11 (2021): e13326, 10.1111/obr.13326. 34423889 PMC9285776

[41] D. G. Marrero , K. N. B. Palmer , E. O. Phillips , K. Miller‐Kovach , G. D. Foster , and C. K. Saha , “Comparison of Commercial and Self‐Initiated Weight Loss Programs in People With Prediabetes: A Randomized Control Trial,” American Journal of Public Health 106, no. 5 (2016): 949–956, 10.2105/AJPH.2015.303035. 26890171 PMC4985082

[42] D. F. Tate , L. D. Lutes , M. Bryant , et al., “Efficacy of a Commercial Weight Management Program Compared With a Do‐It‐Yourself Approach: A Randomized Clinical Trial,” JAMA Network Open 5, no. 8 (2022): e2226561, 10.1001/jamanetworkopen.2022.26561. 35972742 PMC9382439

[43] J. Harvey‐Berino , R. A. Krukowski , P. Buzzell , D. Ogden , J. Skelly , and D. S. West , “The Accuracy of Weight Reported in a Web‐Based Obesity Treatment Program,” Telemedicine Journal and e‐Health 17, no. 9 (2011): 696–699, 10.1089/tmj.2011.0032. 21882997 PMC3241925

[44] A. Berthoumieux , S. Linke , M. Merry , A. Megliola , J. Juusola , and J. Napoleone , “Long‐Term Results of a Digital Diabetes Self‐Management and Education Support Program Among Adults With Type 2 Diabetes: A Retrospective Cohort Study,” Sci Diabetes Self‐Manag Care 50, no. 1 (2024): 19–31, 10.1177/26350106231221456. 38240247

