# Correction to “Long‐Term Weight Loss Outcomes in a Virtual Weight Care Clinic Prescribing a Broad Range of Medications Alongside Behavior Change”

**DOI:** 10.1002/osp4.70067

**Published:** 2025-03-14

Clark JM, Smith BJ, Juusola JL, Kumar RB. Long‐Term Weight Loss Outcomes in a Virtual Weight Care Clinic Prescribing a Broad Range of Medications Alongside Behavior Change. *Obes Sci Pract*. 2025 Jan 8;11(1):e70036.

Figure 4 and a corresponding reference in the Results section have been removed. Figure 4 reported results independently for various medications. These medications, however, were often prescribed for off‐label uses (not FDA‐approved for chronic weight management) in conjunction with other medications shown in the table, and the individual medications should not be understood as independent variables responsible for the results shown.

We apologize for this error.

