# Supplementary material for: Long‐Term Weight Loss Outcomes in a Virtual Weight Care Clinic Prescribing a Broad Range of Medications Alongside Behavior Change
Source: Obes Sci Pract. 2025 Jan 8;11(1):e70036. doi: 10.1002/osp4.70036 (PMC11711220; doi:10.1002/osp4.70036)
Supplement: Supplementary file 1 — Supporting Information S1 [file OSP4-11-e70036-s001.docx]

**Supplemental Tables**

Supplemental Table 1. Baseline demographic and clinical characteristics for the engaged cohorts.

| Member characteristic at baseline | Engaged cohort: Weekly action taken  (n = 4,778) | Engaged cohort: Weekly weight logged  (n = 1,773) |
| --- | --- | --- |
| Age (y), mean (SD) | 45.2 (9.5) | 46.4 (9.3) |
| Biological sex at birth, n (%)       Female       Male       Intersex       Unknown | 4,448 (93.1)  329 (6.9)  0 (0.0)  1 (<0.1) | 1,640 (92.5)  132 (7.4)  0 (0.0)  1 (0.1) |
| Geographical region, n (%)       Northeast       Midwest       South       West       Unknown | 680 (14.2)  1,120 (23.4)  1,786 (37.4)  1,192 (24.9)  0 (0.0) | 245 (13.8)  443 (25.0)  656 (37.0)  429 ( 24.2)  0 (0.0) |
| Weight (kg), mean (SD) | 99.3 (20.5) | 100.0 (20.1) |
| BMI (kg/m^2^), mean (SD) | 36.1 (6.6) | 36.3 (6.6) |
| BMI category, n (%)       Overweight: 25.0-29.9 kg/m^2^       Class 1 obesity: 30.0-34.9 kg/m^2^       Class 2 obesity: 35.0-39.9 kg/m^2^       Class 3 obesity: ≥40.0 kg/m^2^ | 675 (14.1)  1,792 (37.5)  1,190 (24.9)  1,121 (23.5) | 231 (13.0)  658 (37.1)  442 (24.9)  442 (24.9) |
| Comorbidities, n (%)       Anxiety       Arthritis       Body pain       Depression       Dyslipidemia       Hypertension       Metabolic dysfunction-associated steatotic liver disease (MASLD)       Obstructive sleep apnea       Polycystic ovary syndrome (PCOS)       Pre-diabetes       Type II diabetes | 846 (17.7)  985 (20.6)  2,756 (57.7)  1,184 (24.8)  1,077 (22.5)  1,069 (22.4)  348 (7.3)  629 (13.2)  396 (8.3)  509 (10.7)  130 (2.7) | 302 (17.0)  382 (21.5)  1,048 (59.1)  449 (25.3)  424 (23.9)  419 (23.6)  147 (8.3)  247 (13.9)  132 (7.4)  187 (10.5)  49 (2.8) |

BMI = body mass index

SD = standard deviation

Supplemental Table 2. Results of sensitivity analysis using an unadjusted linear mixed-effects model on the full cohort.

| Follow-up time point (n = 66,094) | Coefficient, kg (95% CI) | P-value |
| --- | --- | --- |
| 3 months | -2.9 (-2.9, -2.8) | <0.001 |
| 6 months | -5.2 (-5.2, -5.1) | <0.001 |
| 12 months | -6.5 (-6.6, -6.4) | <0.001 |

CI = confidence interval

Negative coefficients indicate weight loss.

Supplemental Table 3. Baseline demographic and clinical characteristics for subgroups stratified by GLP-1 prescriptions.

| Member characteristic at baseline | ≥6 GLP-1 prescriptions  (n = 265) | No GLP-1 prescriptions  (n = 7,966) | P-value |
| --- | --- | --- | --- |
| Age (y), mean (SD) | 43.9 (9.0) | 44.0 (9.7) | 0.848 |
| Biological sex at birth, n (%)       Female       Male       Unknown | 238 (89.8)  27 (10.2)  0 (0.0) | 7,325 (92.0)  637 (8.0)  4 (0.1) | 0.409 |
| Geographical region, n (%)       Northeast       Midwest       South       West | 69 (26.0)  50 (18.9)  106 (40.0)  40 (15.1) | 1,118 (14.0)  1,767 (22.2)  3,104 (39.0)  1,977 (24.8) | **<0.001** |
| Weight (kg), mean (SD) | 98.2 (17.9) | 98.5 (20.3) | 0.808 |
| BMI (kg/m^2^), mean (SD) | 35.4 (5.9) | 35.7 (6.4) | 0.494 |
| BMI category, n (%)       Overweight: 25.0-29.9 kg/m^2^       Class 1 obesity: 30.0-34.9 kg/m^2^       Class 2 obesity: 35.0-39.9 kg/m^2^       Class 3 obesity: ≥40.0 kg/m^2^ | 39 (14.7)  111 (41.9)  62 (23.4)  53 (20.0) | 1,260 (15.8)  3,061 (38.4)  1,932 (24.3)  1,713 (21.5) | 0.719 |
| Comorbidities, n (%)       Anxiety       Arthritis       Body pain       Depression       Dyslipidemia       Hypertension       Metabolic dysfunction-associated steatotic liver disease (MASLD)       Obstructive sleep apnea       Polycystic ovary syndrome (PCOS)       Pre-diabetes       Type II diabetes | 70 (26.4)  64 (24.2)  163 (61.5)  95 (35.8)  62 (23.4)  66 (24.9)  17 (6.4)  33 (12.5)  32 (12.1)  50 (18.9)  10 (3.8) | 1,450 (18.2)  1,514 (19.0)  4,458 (56.0)  1,933 (24.3)  1,688 (21.2)  1,670 (21.0)  559 (7.0)  1,056 (13.3)  699 (8.8)  852 (10.7)  194 (2.4) | **0.001**  **0.044**  0.084  **<0.001**  0.431  0.141  0.798  0.774  0.080  **<0.001**  0.239 |

BMI = body mass index

GLP-1 = glucagon-like peptide 1 agonist medication

SD = standard deviation

P-values in bold are statistically significant.

Supplemental Table 4. Comparison of baseline demographic and clinical characteristics for members with and without follow-up weight reported.

| Member characteristic at baseline | Follow-up weight reported  (n = 66,094) | Follow-up weight not reported  n = (59,730) | P-value |
| --- | --- | --- | --- |
| Age (y), mean (SD) | 42.6 (9.6) | 41.1 (9.8) | **<0.001** |
| Biological sex at birth, n (%)       Female       Male       Intersex       Unknown | 60,449 (91.5)  5,628 (8.5)  0 (0.0)  9 (<0.1) | 53,593 (89.7)  6,128 (10.3)  2 (<0.1)  15 (<0.1) | **<0.001** |
| Geographical region, n (%)       Northeast       Midwest       South       West       Unknown | 9,040 (13.7)  14,296 (21.6)  26,845 (40.6)  15,908 (24.1)  5 (<0.1) | 8,020 (13.4)  11,770 (19.7)  26,267 (44.0)  13,672 (22.9)  1 (<0.1) | **<0.001** |
| Weight (kg), mean (SD) | 99.6 (20.6) | 100.5 (21.3) | **<0.001** |
| BMI (kg/m^2^) mean (SD) | 36.0 (6.5) | 36.3 (6.7) | **<0.001** |
| BMI category, n (%)       Overweight: 25.0-29.9 kg/m^2^       Class 1 obesity: 30.0-34.9 kg/m^2^       Class 2 obesity: 35.0-39.9 kg/m^2^       Class 3 obesity: ≥40.0 kg/m^2^ | 9,025 (13.7)  25,408 (38.4)  16,786 (25.4)  14,875 ( 22.5) | 7,642 (12.8)  22,453 (37.6)  15,447 (25.9)  14,188 (23.8) | **<0.001** |
| Comorbidities, n (%)       Anxiety       Arthritis       Body pain       Depression       Dyslipidemia       Hypertension       Metabolic dysfunction-associated steatotic liver disease (MASLD)       Obstructive sleep apnea       Polycystic ovary syndrome (PCOS)       Pre-diabetes       Type II diabetes | 14,476 (21.9)  12,117 (18.3)  36,852 (55.8)  17,688 (26.8)  13,765 (20.8)  1,3818 (20.9)  4,918 (7.4)  9,334 (14.1)  6,793 (10.3)  8,064 (12.2)  1,482 (2.2) | 13,142 (22.0)  9,986 (16.7)  32,151 (53.8)  15,227 (25.5)  11,427 (19.1)  12,311 (20.6)  4,607 (7.7)  8,514 (14.3)  6,650 (11.1)  7,195 (12.0)  1,631 (2.7) | 0.673  **<0.001**  **<0.001**  **<0.001**  **<0.001**  0.199  0.070  0.508  **<0.001**  0.405  **<0.001** |

BMI = body mass index

SD = standard deviation

P-values in bold are statistically significant. 
